# 

**Published:** 1916-03-04

**Authors:** 


### CONTENTS.

PAGE

**The Influence of Evidence in Medical Practice** ... ... ... ... ... ... 489

**The Cancer Problem** ... ... ... ... ... ... 490

**Hospital and Institutional News** ... ... ... 491

Lord Knutsford's Accident—The New Swedish Hospital—Dr. Helen Campbell's Salary Withheld—The Supply of Surgical Dressings by Poor-Law Medical Officers—The Springfield Military Hospital—Scotland's Latest Sanatorium—A Tuberculosis Officer for the City of Westminster—The Welsh Medical School—The Chartered Accountant as Secretary—Proposed Reduction of Notification Fees—Are Sanatoria a Waste of Money? The Housing Problem and Tuberculosis—Hospital Officers before the Tribunals—Birmingham Hospitals and the War Office—The Strain on Munition Workers—The New Arbroath Infirmary—War Work at Liverpool Royal Infirmary—Economy in Expensive Drugs—Another Health Expert—A Poor-Law Departure at Leeds—The Economic Aspect of Displacing Attendants by Women—Municipal Management at Bradford: Doctor's Detailed Criticism—"Hospital Shirkers"—Paying Patients in General Wards—Sensation-mongering in the Lay Press—This Week's Drug Market.

**Traumatic Neurasthenia** .. ... ... ... 497

The Borderline of Malingering.

**Vaccines for the Allied Armies** ... ... ... 498

The Inoculation Department at St. Mary's Hospital.

**Hospitals and the Poets** ... ... ... ... 499

V.—Dr. Erasmus Darwin, Scientist, Physician, and Poet.

PAGE

**The Matrons' and Sisters' Department** ... 501

The Analogy Between the Legal and Nursing Professions. By the Hon. Sir Charles Russell, Bart.

Round the Hospitals.

The Matron's Economies—Textile Supplies, Blankets—An Advantage from the War—The Laundry Department—The Spring Cleaning—Better Candidates for Training—Generous Help for the Editor

**The Florence Nightingale Memorial** ... ... 503

**Schools of Massage** ... ... .. ... ... 504

National Hospital for the Paralysed and Epileptic.

**The Institutional Library** ... ... ... ... 505

**Hospital Architecture and Construction** ... 507

Lincoln County Hospital.

**King Edward's Hospital Fund** ... ... ... 507

The Inherent Statistical Difference of Particular Hospitals.

**Textile Supplies for Hospitals** ... ... ... 509

VI.—Hospital Blankets.

**The Middlesex Hospital** ... ... ... ... ... 511

**The London Hospital**... ... ... ... ... 511

**Parliament and the Profession** ... ... ... 512

**The Editor's Letter-Box** ... ... ... ... 513

Domestic Economy in Isolation Hospitals—An Appeal for £200,000.

**Gifts and Bequests** ... ... ... ... ... ... 514

**Passing Events and Topics** ... ... ... ... 514

**The Institutional Worker** ... ... (Suppl.) 1

Economies in the Matron's Department: Answer to February Question.

